# Aggregation‐induced emission luminogens for augmented photosynthesis

**DOI:** 10.1002/EXP.20210053

**Published:** 2022-03-21

**Authors:** Haixiang Liu, Neng Yan, Haotian Bai, Ryan T. K. Kwok, Ben Zhong Tang

**Affiliations:** ^1^ HKUST‐Shenzhen Research Institute Nanshan Shenzhen China; ^2^ Department of Chemical and Biological Engineering Department of Chemistry, The Hong Kong Branch of Chinese National Engineering Research Center for Tissue Restoration and Reconstruction Institute for Advanced Study The Hong Kong University of Science and Technology Kowloon Hong Kong China; ^3^ School of Energy and Environment and State Key Laboratory of Marine Pollution City University of Hong Kong Kowloon Hong Kong China; ^4^ School of Science and Engineering, Shenzhen Key Laboratory of Functional Aggregate Materials The Chinese University of Hong Kong Shenzhen China

**Keywords:** aggregation‐induced emission, carbon neutrality, photosynthesis, sustainable development

## Abstract

Photosynthesis is promising in sequestrating carbon dioxide and providing food and biofuel. Recent findings have shown that luminescent materials could shift the wavelength of light to a more usable range for augmented photosynthesis. Among them, aggregation‐induced emission luminogens (AIEgens) have advantages of efficient light conversion, high biocompatibility, large Stokes’ shift, and so on. In this perspective, emerging reports of augmented photosynthesis with luminescent materials, especially the AIEgens are included. We emphasized the spectra shift characteristics, material formation, and sustainable development based on augmented photosynthesis.

## Graphical abstract

Augmented photosynthesis will increase the amount of CO_2_ sequestration and provide sustainable food and biofuel. Luminescent materials that shift the wavelength to a more usable range could augment photosynthesis. Recent findings of augmented photosynthesis by luminescent materials especially aggregation‐induced emission luminogens were summarized and a bright prospect has been speculated to inspire more research efforts.

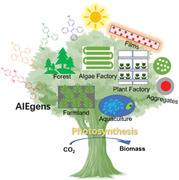

There are emerging findings of augmented photosynthesis with increased efficiency of light usage and productivity by luminescent materials. Among them, aggregation‐induced emission luminogens (AIEgens) have the advantages of efficient light conversion in aggregate state, high biocompatibility, large Stokes’ shift and so on,^[^ ^1^, ^2^ ^]^ thus they are promising as spectra shift materials.^[^ ^3^ ^]^ In this perspective, the proceedings of pilot explorations of luminescent materials for augmented photosynthesis will be included. The possible future directions of this interdisciplinary field will also be speculated to inspire more research efforts.

## 1 PHOTOSYNTHESIS AND SUSTAINABLE DEVELOPMENT

It has reached a consensus that the elevation of global temperature, if not controlled now, would lead to severe damage to the world.^[^ ^4^, ^5^ ^]^ The greenhouse gas emissions have been recognized as the main contributor to global warming and climate change, becoming a big concern for urban densification.^[^ ^6^ ^]^ Since industry age, the atmospheric concentration of carbon dioxide has increased from 280 to 410 ppm due to huge emissions. In 2019, about 36 billion metric tons of carbon dioxide were emitted into atmosphere. It has been estimated that around 70% of the global greenhouse gas emissions are made by the cities, which are mainly used for the energy supply sector. Recently, numerous countries have committed to reaching carbon neutrality (net‐zero carbon dioxide emissions) within a short time. For instance, in September 2020, China proposed to peak carbon dioxide emissions before 2030 and realize carbon neutrality by 2060.^[^ ^7^ ^]^ In October 2020, Japan and South Korea committed to reaching carbon neutrality by 2050.^[^ ^8^, ^9^ ^]^ Currently five EU countries have set the target of climate neutrality in law: Sweden aims to reach net‐zero emissions by 2045 and Denmark, France, Germany, and Hungary by 2050.^[^ ^10^ ^]^ Along with the climate changes, there are also grand challenges of food and energy shortage. The global population is projected to be about 10 billion in 2050, doubling in the energy and food requirement.^[^ ^11^ ^]^ The lack of renewable energy and food will be a catastrophe in the future. Therefore, achieving carbon neutrality as well as increasing food and energy supply are of most importance now.

**FIGURE 1:**
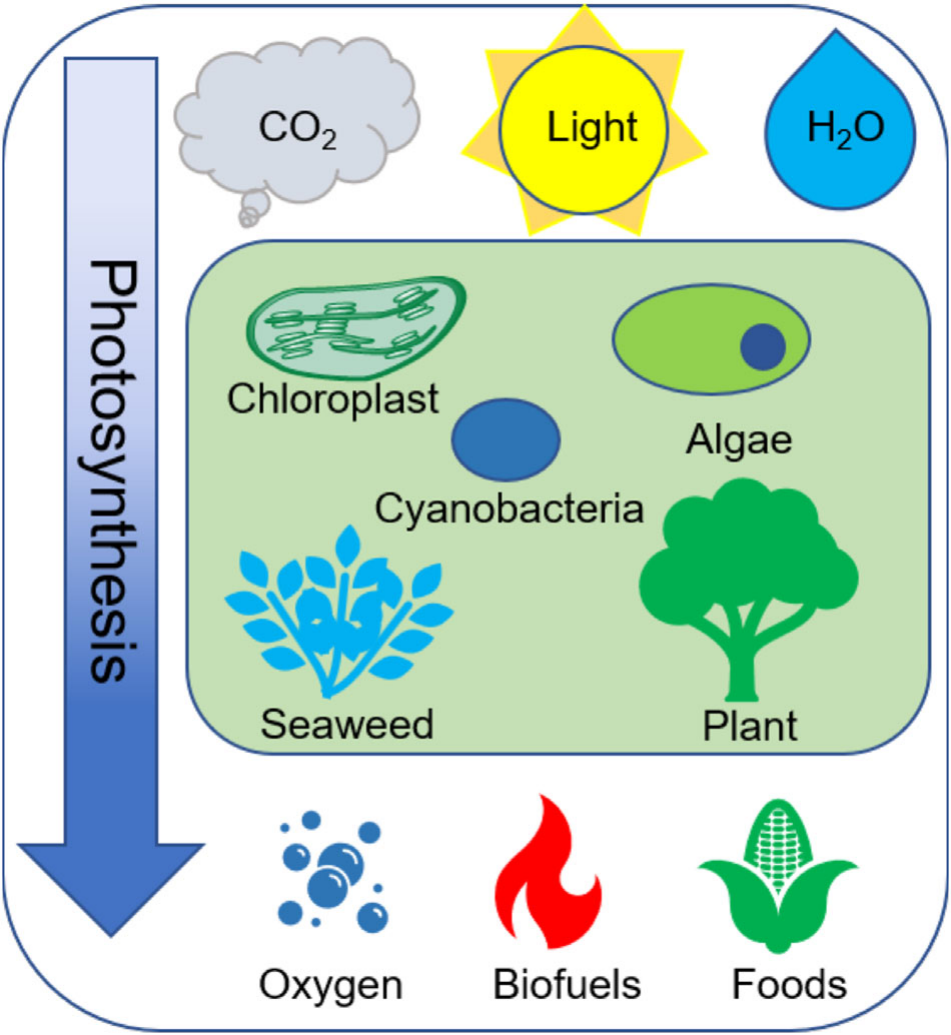
Photosynthesis promotes sustainable development. Photosynthetic units utilize light energy to convert carbon dioxide and water into oxygen and biomass which provides oxygen, sustainable foods, and biofuel

Natural photosynthesis could covert inorganic carbon (mainly carbon dioxide) into biomass containing sugars and lipids using photon energy (Figure 1). The photosynthesis of plants, algae, and photosynthetic bacteria could fix huge amounts of carbon dioxide and maintain the balance of “carbon cycle.”^[^ ^12^ ^]^ And to date, photosynthetic organisms remain to be the best option for the sustainable production of food and biofuel. For example, microalgae have been developed to produce third‐generation biofuel because the carbon dioxide emitted is equal to that sequestrated using microalgae biofuel.^[^ ^13^ ^]^ Increasing the quantities of photosynthetic organisms will largely consume the carbon dioxide and provide plenty of food and biofuel. However, due to the loss of forest, land desertification, marine pollution, low productivity, and high cost, expanding these organisms in natural habitats is limited.^[^ ^14^, ^15^, ^16^ ^]^ Instead, improving the photosynthetic efficiency and the biomass productivity could be an alternative choice.^[^ ^17^ ^]^ Furthermore, building plant and microalgae factories might give rise to a green industry that can utilize the previously useless land such as desert.

## 2 EFFICIENCY OF LIGHT USAGE IN PHOTOSYNTHESIS

**FIGURE 2:**
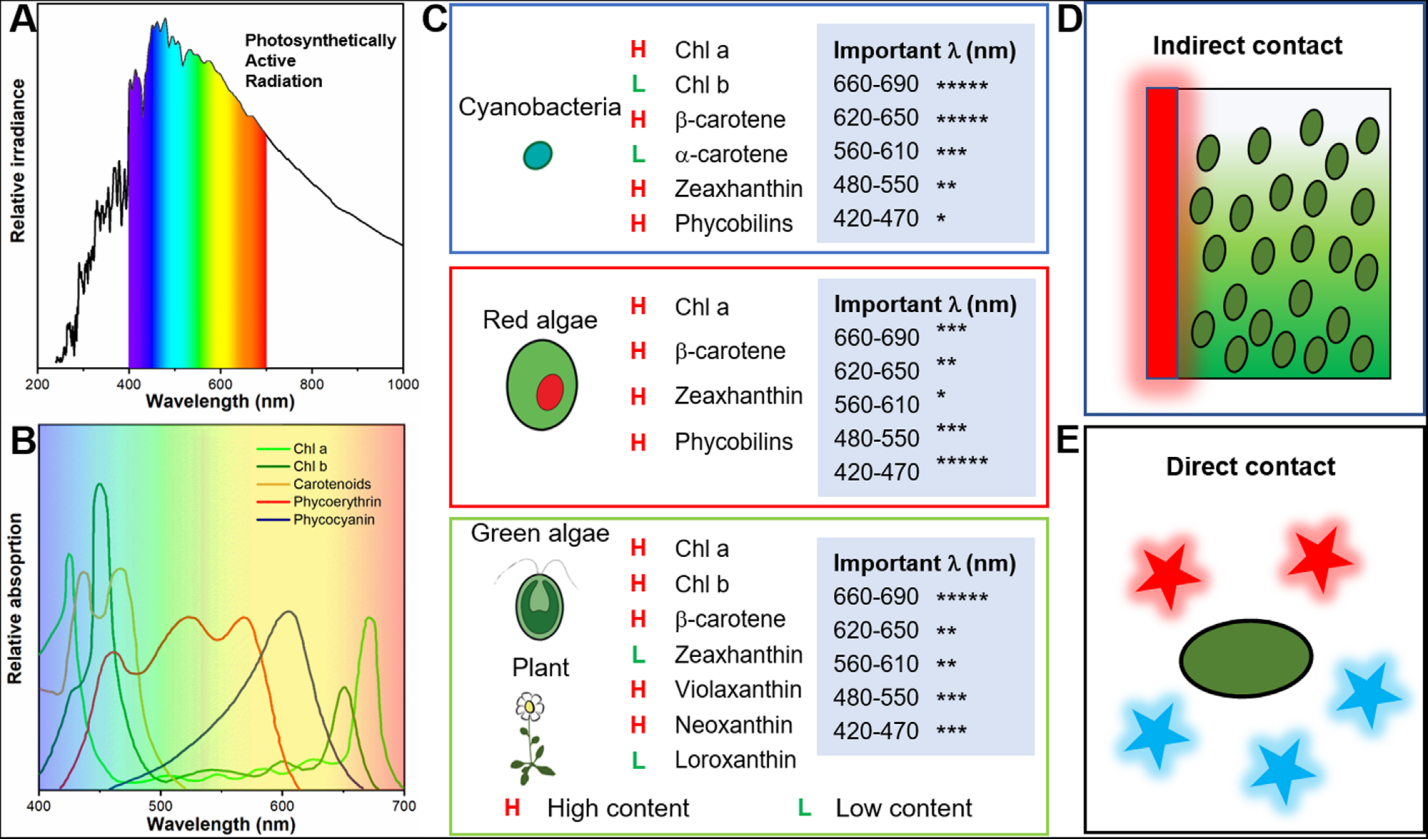
Light utilization by photosynthetic units. (A) the photosynthetically active radiation (PAR) in the typical solar spectra, (B) the relative absorption of common pigments of photosynthetic units. Reproduced with permission.^[^ ^30^ ^]^ Copyright 2020, American Chemical Society, (C) the profile of pigments in photosynthetic units and the related important wavelength of photosynthesis, and augmented photosynthesis by (D) indirect contact and (E) indirect contact. Reproduced with permission.^[^ ^19^ ^]^ Copyright 2014, Elsevier

Increasing the coverage of photosynthetic organisms and the efficiency of photosynthesis contribute to the reduction of atmospheric carbon dioxide. Unfortunately, photosynthetic efficiency of plants and algae in natural condition is quite low largely due to the inability of using a large portion of photons. Only about 5% of solar energy reached on the leaf surface could be transformed into biomass. One reason is that photosynthetically active radiation (PAR) covers only 400 to 700 nm in light spectrum (Figure 2A).^[^ ^12^ ^]^ It only accounts for about 43% of the typical sunlight irradiation reaching the earth surface, which means that over half of the sunlight is wasted for the photosynthesis process. Thus, extending the absorption range outside PAR in both ultraviolet (UV) and infrared will provide more energy for photosynthesis. Moreover, only a small portion inside PAR can be used with high efficiency. This further decreased the efficiency of photosynthesis under natural condition. The efficiency of light usage is mainly determined by the absorption of the pigments inside (Figure 2B). Those typical pigments include chlorophyl a (maximal absorption peaks, around 430 and 670 nm), chlorophyl b (maximal absorption peaks, around 450 and 650 nm), carotenoids (maximal absorption peak, around 470 nm), and phycobilin (maximal absorption peaks, around 615 nm), etc. Among those different pigments, chlorophyl is widely presented in almost all photosynthetic organisms from cyanobacteria to algae and plants, and is vital in the light reaction of photosystems. It has main absorption in the blue (around 425–475 nm) and red region (around 650–675 nm). The red lights excite chlorophyl to the lower excited state, while blue lights excite that to higher excited state. The photophysical process from higher excited state to the lower excited state might generate excess heat, which affects the proper function of photosystem and decreases the photosynthesis speed. Apart from chlorophyl, carotenes, and phycobilin absorb light and transfer energy to chlorophyl.^[^ ^18^ ^]^ The preferences of different wavelengths for photosynthetic organisms are summarized in Figure 2C. For cyanobacteria, it can efficiently utilize green, yellow, and red light, but to a less content of blue light. For red algae with high content of phycoerythrin, it absorbs strongly in blue regions. For green algae and land plants, red and blue lights are mostly absorbed.^[^ ^19^ ^]^ Accordingly, using light source with specific wavelengths could (1) provide more energy that can be efficiently converted by photosynthesis and (2) reduce the harmful irradiation and excess energy during photosynthesis. Therefore, optimizing wavelength of light source could increase photosynthetic efficiency.

## 3 SPECTRA SHIFT OF LUMINESCENT MATERIALS FOR AUGMENTED PHOTOSYNTHESIS

Photosynthesis highly depends on the light intensity and their spectrum (UV, blue, green, yellow, red, or even near‐infrared). The light sources can be roughly separated into two categories: (1) sunlight in the natural environment which can be easily obtained and (2) artificial light in factories which can be made with different purpose. However, the sunlight contains full spectra of light and the commercially used cool white LED contains a large portion of blue and yellow lights. The spectra between light sources and photosynthesis are not best matched. Therefore, light utilization has been one of the major limitations for boosting the photosynthesis of photosynthetic organisms using current light sources. However, construction of specific light source for each of the photosynthetic species is tedious and not cost‐effective. Instead, spectra shift from unutilized wavelength to PAR as well as lower efficiency to higher efficiency could increase the photosynthetic speed and yield.^[^ ^20^ ^]^ Luminescent materials such as laser dyes, organic fluorescent molecules, phosphors, and conjugated polymers, could absorb certain wavelength light and emit another wavelength light, which might shift the wavelength to a more usable range. The reports of augmented photosynthesis by luminescent materials are summarized in Table 1. In 1984, laser dyes have been utilized for enhancing microalgae growth as an indirect layer between algae and light source. While direct adding laser dyes did not induce the increase in growth.^[^ ^21^ ^]^ Since then, fluorescent coating with different materials has become the popular way of spectra shift for microalgae and plants (Figure 2D).^[^ ^22^, ^23^, ^24^, ^25^ ^]^ Recently, conjugated polymers have also been used as direct contact with chloroplast, photosynthetic bacteria, algae, and plants with high photosynthetic enhancement (Figure 2E).^[^ ^26^, ^27^, ^28^, ^29^ ^]^ Future investigations of those materials for stability, fate, and interaction with photosynthetic organisms will be needed.

**TABLE 1:** Augmented photosynthesis by luminescent materials

**Materials**	**Spectral shift**	**Methods**	**Species**	**Enhancement**	**Ref**.
Laser dyes	Green to red	Indirect contact	Green alga	19% for biomass	^[^ ^21^ ^]^
Phosphor	UV/green to red	Indirect contact	Red alga	36% for biomass	^[^ ^22^ ^]^
Uvitex OB	UV‐A to blue	Indirect contact	Cyanobacterium	38% for biomass	^[^ ^23^ ^]^
Lumogen F Red 305	Green to red	Indirect contact	Plant	18.7% for biomass	^[^ ^25^ ^]^
Conjugated polymers	UV to blue/green	Direct contact	Chloroplast	∼90% for ATP	^[^ ^26^ ^]^
Conjugated polymers	UV to blue, UV/blue to green, UV/blue to red	Direct contact	Bacterium	49.56% for ATP 37.73% for ATP 29.35% for ATP	^[^ ^27^ ^]^
Conjugated polymers	Green to far‐red	Direct contact	Green alga Plant	76% for NADPH 97% for ATP	^[^ ^28^ ^]^
AIEgens	Green to red	Indirect contact	Green alga	26% for biomass	^[^ ^30^ ^]^
AIEgens	UV to blue, green to red	Direct contact	Chloroplast	54% for ATP 33% for ATP	^[^ ^32^ ^]^
AIEgens	UV/blue to green	Direct contact	Cyanobacterium	500% for biomass	^[^ ^31^ ^]^

## 4 AUGMENTED PHOTOSYNTHESIS BY AIEGENS

Conventional fluorophores suffer from aggregation caused quenching effect (ACQ), where the fluorescence will be quenched in high concentration or the aggregate state. This limits the usage of nanoparticle form and the high concentration in film doping, leading to limited spectra shift and poor stability. On the contrary, aggregation‐induced emission luminogens (AIEgens) are highly emissive in aggregate state, making it promising in fluorescent films and nanoparticles. AIEgens have also been utilized in spectra shift for augmented photosynthesis.

**FIGURE 3:**
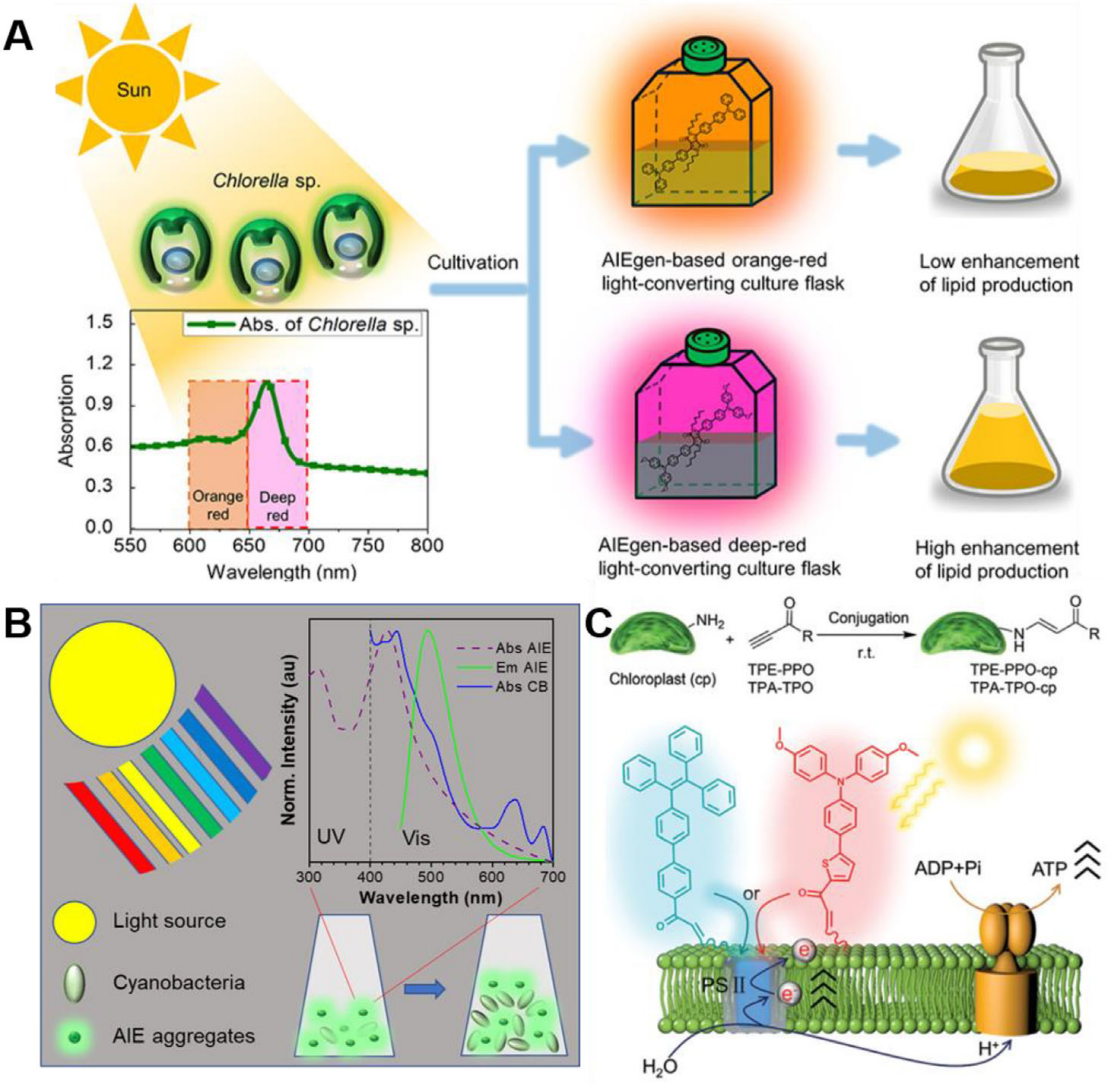
Augmented photosynthesis by AIEgens. (A) augmented growth and lipid production of green algae by culture flask with AIEgens based conversion layer. Reproduced with permission.^[^ ^30^ ^]^ Copyright 2020, American Chemical Society; (B) boosted growth of cyanobacteria by directly adding AIE aggregates in the culture medium. Reproduced with permission.^[^ ^31^ ^]^ Copyright 2021, American Chemical Society; and (C) augmented photosynthesis of chloroplast by direct bioconjugation of AIEgens. Reproduced with permission.^[^ ^32^ ^]^ Copyright 2021, The Royal Society of Chemistry

AIEgens doped film has been utilized in culturing green algae (*Chlorella* sp.), which increased the biomass and lipid content (Figure 3A).^[^ ^30^ ^]^ AIE active diketopyrrolopyrroles (DPP) were fabricated with polymethyl methacrylate (PMMA) to generate fluorescent film with spectra shift of green to red. The obtained AIEgens 3‐(4′‐(bis(4‐methoxyphenyl)amino)‐[1,1′‐biphenyl]‐4‐yl)‐6‐(4‐bromophenyl)‐2,5 dihexyl‐2,5‐dihydropyrrolopyrrole‐1,4‐dione (M2) had an absorption peak of 515 nm and an emission peak around 646 nm and a large Stokes’ shift of 131 nm. It had a pretty high fluorescent quantum yield in PMMA film of 11–13%. Applying M2 containing PMMA film on the front cover of culturing flask increased the photosynthetic photon flux density of orange red (600–650 nm) by 4% and deep red (650–700 nm) by 3.4%. This led to the enhancement of biomass by 26% and total fatty acid methyl esters by 28.8%. In this work, AIEgens were doped in a PMMA film that stuck to the outside of the culturing flask. It was easy handling and avoided direct contact with microalgae. However, due to the isotropic nature of fluorescence and the absorption and scattering of PMMA film, a large portion of photons in the light source have been wasted, resulting in limited light utilization.

AIEgens could also be directly added into the culture medium of microalgae to shift the light spectra as well as change the light distribution inside the medium (Figure 3B).^[^ ^31^ ^]^ Two AIEgens 3‐diphenylamino‐6‐(2‐pyridinyl)phenyldiphenylboron (TPBA) and 4‐((2,2‐difluoro‐5‐phenyl‐2,3‐dihydro‐1,3,4,2‐oxadiazaborol‐3‐ylidene)methyl)‐*N*,*N*‐dimethylaniline (APO) have been utilized to boost the growth of cyanobacteria (*Synochococus baciliaris*). These two AIEgens absorbed UV/blue lights and emitted green and yellow lights, which could be more efficiently used by cyanobacteria in the aspect of wavelength. In the meantime, AIEgens formed nano sized aggregates in the culture medium of cyanobacteria, which served as numerous nano lights. The photosynthetic parameters were increased by adding AIEgens, including the maximum photosynthetic quantum yield, the effective photochemical quantum yield of photosystem II, and the relative electron transport rate. The biomass and lipid were enhanced five‐fold compared to control after 14 days culturing. Furthermore, AIEgens showed excellent protection effect under UV light irradiation. In this work, AIEgens were utilized as aggregates inside the culture medium, which reduced the loss of photons in light propagation. However, the recycling of AIEgens is needed in sustainable production.

The distance between AIEgens and the photosynthetic units could be even shorter by direct bioconjugation (Figure 3C).^[^ ^32^ ^]^ AIEgens 1‐(4′‐(1,2,2‐triphenylvinyl)‐[1,1′‐biphenyl]‐4‐yl)prop‐2‐yn‐1‐one (TPE‐PPO) and 1‐(4‐(4‐(bis(4‐methoxyphenyl)amino)phenyl)cyclopenta‐1,3‐dien‐1‐yl)prop‐2‐yn‐1‐one (TPA‐TPO) with activated alkene have been conjugated to chloroplast by click reaction. The reaction happened between AIEgens and proteins on the chloroplast surface to form covalent bonding. It showed that the normal function of chloroplast was seldomly affected by the addition of AIEgens at low concentration. The spectra shift of UV to blue and green to red by TPE‐PPO and TPA‐TPO increased adenosine triphosphate (ATP) generation under UV light and white light by 54% and 33%, respectively. In this work, AIEgens were directly attached to the surface of photosynthetic units, which had the highest light utilization during propagation. However, the procedure is relatively complicated and not suitable for microorganisms that divide fast to generate new cells. On the contrary, conjugating AIEgens to the leaves of plants might have stable and long‐term improvement of photosynthesis and growth.

## 5 CONCLUSION AND PROSPECT

Augmented photosynthesis toward sustainable development is an interdisciplinary field involving physics, chemistry, biology, materials, engineering, and so on. It is a promising but young field with some emerging findings demonstrating the feasibility of using AIEgens for efficient spectra shift to increase the portion of photosynthetically efficient irradiation. However, there is still a long journey to go in order to make a real difference.

First, the efficiency of spectra shift highly depends on the photophysical properties of AIEgens. They need to have high molar absorptivity and photoluminescence quantum yield, endowing the most efficient conversion. They need to have a large Stokes’ shift, reducing the self‐absorption as much as possible. They need to be highly stable and resistant to photoirradiation, keeping emissive during the long growth period of photosynthetic organisms. By restriction of the molecular motion in the aggregate state, the quantum yield of AIEgens could be as high as 100%.^[^ ^33^ ^]^ Construction of the non‐planar structure and election doner and acceptor of AIE molecule, the Stokes’ shift could reach 200 nm.^[^ ^34^ ^]^ Using higher amount of AIEgens will increase the photostability while not reduce the luminescence.

Apart from the intrinsic efficiency of spectra shift, the formation of the materials also influences the external efficiency. For indirect contact, controlling photon loss during light propagation is important. The absorption and diffraction of light by conversion layer should be minimized. Furthermore, handling the isotropic nature of photoluminescence is needed such as choosing the position of conversion layer. In the case of front side, light will be reduced after passing through conversion film. In the case of backside, light might be largely absorbed and scattered by photosynthetic species.^[^ ^22^ ^]^ On the other hand, construction of light extraction film with higher portion of usable photons after propagation is also promising.^[^ ^25^ ^]^ AIEgens could be easily incorporated into optical polymers such as PMMA and this is suitable for indirect contact. Conventional fluorophores were used at low concentration in film doping such as 0.1% weight of Lumogen F Red 305 to PMMA.^[^ ^25^ ^]^ In contrast, AIEgens could be used at high concentration in film doping, improving the spectra shift effect and photostability.

For direct contact, the light propagation issue is largely relived, but the interaction and recycle as well as the biocompatibility are important. However, some luminescent materials such as coumarin derivatives^[^ ^21^ ^]^ and photoluminescent phosphor (Ca_0.59_Sr_0.40_Eu_0.01_S)^[^ ^22^ ^]^ are not suitable. In contrast, AIEgens showed excellent biocompatibility to chloroplast, algae, and plants.^[^ ^35^ ^]^ In aquatic microalgae, aggregate formation is possible for light utilization in both spectra shift and spatial redistribution. AIE aggregates could be easily constructed by directly adding into poor solvent, encapsulation in polymetric nanoparticles, and so on. While keeping aggregates outside microalgae and efficiently recycling them is needed. In land plants, stable connection to leaves is suitable for efficient and long‐term enhancement. There are emerging “click reactions” that can be efficiently performed in mild conditions. The method of constructing bioconjugation with plants, especially in large quantities is needed.

The real practice to achieve carbon neutrality needs to efficiently capture and utilize carbon dioxide in large amount.^[^ ^36^, ^37^, ^38^ ^]^ Alternatives could be forest with conjugation of AIEgens, farmland, and greenhouses covered with AIEgens based film; microalgae factories with AIE aggregates; plant factories with AIEgen based OLEDs powered by photovoltaics; green architectures with photosynthetic units, etc. The concentration of carbon dioxide in atmosphere and water is lower than optimal utilization. Increasing the supply of carbon dioxide to photosynthetic plants, algae, and bacteria is also needed. The carbon dioxide sequestration could happen in a large area of forest, farmland, or aquaculture, or a giant architecture of plant and algae factories taking the advantage of vertical space. These land and architectures will become green machines to turn carbon dioxide into food and biofuel. In the end, this green revolution might relieve the global challenges and provide plenty amount of sustainable food and energy.

## CONFLICT OF INTEREST

Ben Zhong Tang is a member of the *Exploration* editorial board. The authors declare no conflict of interest.
